# The more you look, the more you find: challenging results on FDG-PET CT in a patient with neurofibromatosis type I

**DOI:** 10.1186/1471-2342-14-19

**Published:** 2014-05-29

**Authors:** Patrick Schöffski, Christophe M Deroose, Olivier Gheysens, Nele Lips, Koen Slabbaert, Ben Vancleynenbreugel, Philippe Moerman, Willy E Peetermans

**Affiliations:** 1 General Medical Oncology, UZ Leuven, Leuven, Belgium; 2 Nuclear Medicine, UZ Leuven, Leuven, Belgium; 3 Urology, UZ Leuven, Leuven, Belgium; 4 Pathology, UZ Leuven, Leuven, Belgium; 5 General Internal Medicine, UZ Leuven, Leuven, Belgium; 6 Department of General Medical Oncology, University Hospitals Leuven, Laboratory of Experimental Oncology, Division of Oncology, Leuven Cancer Institute, Catholic University Leuven, Herestraat 49, Leuven B-3000, Belgium

**Keywords:** Syphilis, PET, Fluorodeoyglucose, Neurofibromatosis, Testicular seminoma

## Abstract

**Background:**

FDG-PET/CT is part of the standard diagnostic management of a patients with a large variety of common and less common malignant tumors, based on the increased glucose metabolism within tumors.

**Case presentation:**

A hybrid fluorodeoxyglucose positron emission tomography and computed tomography (FDG-PET/CT) was performed in a neurofibromatosis patient to rule out relapse of malignant peripheral nerve sheet tumor. The scan revealed non-malignant neurofibromas, a testis seminoma and hypermetabolic syphilitic granulomata.

**Conclusion:**

This case stresses the need to rule out infectious diseases when atypical hypermetabolic lesions are present.

## Background

FDG-PET/CT is part of the standard diagnostic management of a patients with a large variety of common and less common malignant tumors, based on the increased glucose metabolism within tumors. It is useful for detecting malignant transformation of neurofibromas to MPNST, as malignant MPNST is characterized by higher FDG uptake. A cut-off maximum standardized uptake value (SUV_max_) of 3.5 discriminates neurofibroma from MPNST (sensitivity: 89%; specificity 95%) [1]. High FDG uptake can be seen in non-neoplastic tissues, for instance in inflammatory or infectious lesions. Interpretation of FDG-PET images should thus be done with full knowledge of the case record of the patient.

## Case presentation

**Figure 1:**
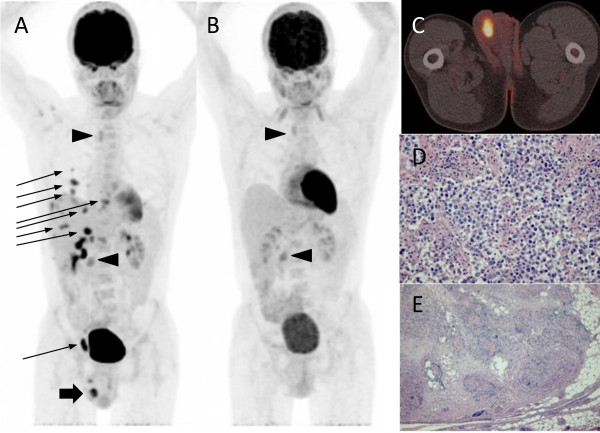
**FDG_PET/C T findings. (A)** FDG-PET/CT performed at diagnosis (large arrow: hypermetabolic seminoma testis; arrowhead: low FDG-uptake in neurofibroma; small arrow: syphilitic granulomatous lesions); **(B)** FDG-PET/CT after orchidectomy, diagnostic laparoscopy and two week treatment with doxocycline, showing persistant uptake only in neurofibromas (arrowheads); **(C)** PET/CT fusion of primary seminoma; **(D)** hematoxylin/eosin stain of seminoma; and **(E)** inguinal granuloma.

A 32 year-old homosexual Caucasian male with neurofibromatosis type I (NF1), café-au-lait patches and known superficial and deep neurofibromas consulted us for unexplained fatigue and weight loss. History and biochemistry were unremarkable. His clinical examination revealed known signs of NF1 and a nodular appearance of his right testicle.FDG-PET/CT was performed to exclude the presence of a NF1-related malignancy (e.g., malignant peripheral nerve sheet tumor, MPNST). It revealed multiple hypermetabolic lesions in the following sites: right scrotum, omentum, peritoneum, neuroforamen C5/C6, sternum, ribs, both lungs and mediastinum (Figure 1). Based on these findings testicular cancer with abdominal, pulmonary and bone involvement had to be ruled out. Scrotal ultrasound showed a multinodular mass in the right testicle and ipsilateral hypoechogenic inguinal nodules. Serum tumor markers were normal.

The patient underwent a simultaneous orchiectomy and diagnostic laparoscopic resection of inguinal and omental lesions. Histology revealed a 2.6 cm testicular seminoma with epididymal invasion. The surrounding stroma, funiculus spermaticus and resected omental and inguinal lesions showed a lymphocytic and granulomatous infiltrate, which was initially interpreted as a sarcoid-like reaction or sarcoidosis. There was no evidence for neurofibroma or sarcoma in any of the tissue samples.

A broad laboratory investigation was performed to rule out some common causes of granulomata. Serologies for *Bartonella*, *Brucella*, *Coxiella*, *Borellia*, *Toxoplasma*, hepatitis B/C, HIV and HTLV-1 were normal. Tuberculosis was ruled out by specific staining. Serology for *Treponema pallidum* was positive (*Treponema pallidum* specific antibody test+, rapid plasma reagin test+, titer 1/16). In retrospect, the patient had no history of urogenital complaints or ulcus durum, rash or arthralgia.

We thus interpreted the hypermetabolic granulomatous lesions as active syphilis (primo-infection), for which antibiotic treatment of both the patient (doxycycline 200 mg/day p.o. for 2 weeks because of an IgE-mediated allergy to penicillin) and his serologically-negative partner was initiated. To confirm the working hypothesis of metabolically active syphilis and to rule out distant spread of seminoma, FDG-PET/CT was repeated 21 days after end of therapy, showing complete regression of all hypermetabolic foci, except for known sites of neurofibroma.. We proposed watchful waiting, adjuvant radiotherapy or systemic chemotherapy as options for stage I seminoma, and the patient opted for adjuvant carboplatin AUC 7. He is currently in three-monthly follow-up without clinical, serological or radiological signs of relapse of testicular cancer progression of NF1-related conditions or active syphilis.

FDG-PET/CT is increasingly used as whole body imaging modality in oncology, with high sensitivity and specificity for a number of malignancies. It is useful for detecting malignant transformation of neurofibromas to MPNST, as malignant MPNST is characterized by higher FDG uptake. A cut-off maximum standardized uptake value (SUV_max_) of 3.5 discriminates neurofibroma from MPNST (sensitivity: 89%; specificity 95%)^1^. Seminoma testis is highly FDG-avid (average SUV_mean_ 9.2) [2]. FDG-PET/CT is able to detect seminomatous lesions, is generally not recommended for seminoma staging [3] but is useful for characterization of residual masses after chemotherapy (sensitivity: 80%; specificity 100%) [4].

The experience with FDG-PET/CT in syphilis is very limited but hypermetabolic syphilitic lesions in bone, lymph nodes, lung, aorta [5], rectum and brain have been reported only in individual patients. In patients presenting with multiple pathologies quantitative evaluation of the FDG PET images might be of use to ascertain the presence of multiple synchronous diseases. One interesting study [6] proposed a cut off value of 40% for the difference between two lesions to discriminate metastases from a synchronous secondary primary tumor. When applying this treshold to this patient this suggests that the neurofibromas are not related to the other foci but it does not suggest that the granulomatous lesions are not related to the testis tumor, which is similar to the visual interpretation of the data. Only 3 cases have been described in the literature with a testicular tumor in a NF-1 patient, so occurrence of seminoma in NF1 patients has to be regarded as fortuitous [7-9].

## Conclusion

FDG-PET/CT detected an incidental seminoma testis in a NF1-patient with simultaneous hypermetabolic thoraco-abdomino-pelvic lesions, which were first attributed to the seminoma, then considered to be sarcoid-like lesions according to histology, and were ultimately diagnosed as syphilitic granulomas based on serology and functional imaging before and after antibiotic treatment. FDG-PET/CT cannot differentiate between active inflammation (infectious or not) and malignancy, so metabolic findings always have to be interpreted with caution and in the context of additional clinical information, as shown in our complex case. Infectious diseases should be ruled out in cases with aspecific pathological findings.

## Consent

Written informed consent was obtained from the patient for publication of this Case report and any accompanying images. A copy of the written consent is available for review by the Editor of this journal.

## Competing interests

The authors declare that they have no competing interests.

## Authors’ contributions

SP and WEP were the responsible treating physicians of the patient. SP and CMD were responsible for the drafting of the manuscript and creation of the figure. CMD, OG and NL were responsible for the execution and interpretation of the PET scans. KS and BVC performed surgery on the patient. PM was responsible for the pathological diagnoses in the patient. All authors gave critical input to the manuscript and consented with its final version. All authors read and approved the final manuscript.

## Pre-publication history

The pre-publication history for this paper can be accessed here:

http://www.biomedcentral.com/1471-2342/14/19/prepub
